# *Vibrio alginolyticus* Reprograms CIK Cell Metabolism via T3SS Effector VopS to Promote Host Cell Ferroptosis

**DOI:** 10.3390/ani14223250

**Published:** 2024-11-13

**Authors:** Weijie Zhang, Chao Huang, Zhihang Chen, Dawei Song, Yujia Zhang, Shuai Yang, Na Wang, Jichang Jian, Huanying Pang

**Affiliations:** 1 Fisheries College, Guangdong Ocean University, Zhanjiang 524025, China; zhangweijie31@stu.gdou.edu.cn (W.Z.); 18350918317@163.com (C.H.); czh14754984647@163.com (Z.C.); 03059@zjhu.edu.cn (D.S.); zhangyujia11@stu.gdou.edu.cn (Y.Z.); yangshuai1@stu.gdou.edu.cn (S.Y.); jianjc@gmail.com (J.J.); 2 Guangdong Provincial Key Laboratory of Aquatic Animal Disease Control and Healthy Culture & Key Laboratory of Control for Diseases of Aquatic Economic Animals of Guangdong Higher Education Institutes, Zhanjiang 524025, China; 3 Chinese Academy of Inspection and Quarantine, Beijing 100176, China; wangna85@yeah.net

**Keywords:** *Vibrio alginolyticus*, T3SS, *vopS*, metabolomics, ferroptosis

## Abstract

*Vibrio alginolyticus* is a Gram-negative pathogen of both marine animals and humans, resulting in significant losses for the aquaculture industry. Emerging evidence indicates that *V. alginolyticus* manipulates cell death for its pathogenicity, but the underlying molecular mechanisms remain unclear. Here, a gene designated *vopS* in *V. alginolyticus* HY9901 was identified, which was predicted to encode the T3SS effector protein. To determine whether VopS contributes to the pathogenesis of *V. alginolyticus*, the Δ*vopS* mutant strain was constructed and phenotypically characterized. The deletion of VopS not only reduced the ability to secrete extracellular proteases and virulence but also affected the expression of the T3SS genes. Furthermore, VopS was cytotoxic and induced apoptosis, as confirmed by elevated LDH and the activation of caspase-3. Metabolomic analysis revealed considerable metabolomic disruptions upon *V. alginolyticus* infection. The VopS effector induced host cell ferroptosis by promoting the synthesis of adrenic acid, depleting cellular glutathione, and subsequently increasing the accumulation of ferrous (Fe^2+^). Taken together, our findings provide that the VopS effector is an essential virulence factor of *V. alginolyticus*, which can lead to ferroptosis.

## Simple Summary

Type III secretion system (T3SS) is an essential virulence determinant for *Vibrio alginolyticus.* In this study, we highlighted the significance of T3SS effector protein VopS in the *V. alginolyticus* infection. The *vopS* gene, which can promote the virulence of *V. alginolyticus* to Epinephelus coioides, is required for extracellular protease secretion. Also, it was demonstrated that VopS was involved not only in *V. alginolyticus*-mediated apoptosis but also in ferroptosis. Collectively, we identified that the VopS effector is a metabolic disruptor, promoting host cell ferroptosis.

## 1. Introduction

*Vibrio alginolyticus* is a Gram-negative bacterium distributed in estuarine and marine environments [1]. Obviously, *V. alginolyticus* causes vibriosis in substantial fish and shrimp species, including *Sparus aurata* L. [2], orange-spotted grouper (*Epinephelus coioides*) [3], *Penaeus vannamei* [4], and kuruma prawn (*Penaeus japonicus*) [5], leading to high mortalities and severe economic losses in aquaculture worldwide. It is also a causative agent of acute gastroenteritis in humans linked to consuming water and raw seafood. Occasionally, *V. alginolyticus* induces wound infection and sepsis in immunocompromised individuals [6]. Consequently, it is critical to comprehend the pathophysiology of *V. alginolyticus* and generate a vaccine that effectively prevents vibriosis.

Type III secretion system (T3SS) is an essential virulence determinant for Gram-negative pathogenic bacteria, such as *Shigella* spp. [7], *Salmonella* spp. [8], and *Yersinia* spp. [9]. It is reported that T3SS directly delivers effector proteins into host cells, triggering host-cell death and manipulating the innate and adaptive immune system [10,11]. Although the T3SS machinery is usually conserved among Gram-negative bacteria, the effectors’ functions vary greatly. Although comparative genomic research showed that the T3SS of *Vibrio harveyi* and *V. alginolyticus* are comparable, little is known about the effectors of *V. alginolyticus*. Thus, functional characterization of T3SS effectors is essential.

To date, four T3SS effectors have been identified, including VopQ, VopR, VopS, and VPA0450 [12,13]. In particular, the VopS effector has been extensively studied and found to contribute to pathogenesis as a bacterial AMPylator [14]. After entering the host cell, VopS from *V. parahaemolyticus* AMPylates a threonine residue in the switch I region of Rho GTPases by Fic (filamentation induced by cAMP) domains. This acylation of Rho GTPases prevents their interaction with downstream effectors, even leading to cell damage and cell death [15]. It is also reported that VopS interferes with the activation of the host NLRC4 (NOD-like receptor CARD domain-containing 4) inflammasome during *V. parahaemolyticus* infection [16]. Although extensive research has been conducted, the mechanism of VopS in *V. alginolyticus* is unclear.

Ferroptosis is a recently discovered iron-dependent regulated cell death manifested by intracellular phospholipid peroxidation [17]. It is known that T3SS plays important roles in regulating host cellular physiological processes, but its regulatory roles and mechanisms in host ferroptosis remain elusive. Here, an in-frame deletion of *vopS* was performed, and then the physiology and pathogenicity of ∆ *vopS* were determined. Also, we analyzed the metabolic reprogramming of the CIK cells during *V. alginolyticus* infection and the role of the VopS effector of T3SS in modulating host cell metabolism using the LC–MS/MS system. Collectively, we identified that the VopS effector is a metabolic disruptor, promoting host cell ferroptosis.

## 2. Materials and Methods

### 2.1. Bacterial Strains and Culture Conditions

*Vibrio alginolyticus* HY9901 was stored in our laboratory and used as the wild-type strain throughout this study [1]. The *V. alginolyticus* HY9901 Δ*vopS* mutant strain was generated using double-crossover homologous recombination [18]. The strains, vectors, and primer sequences used to generate the strains are listed in Table 1 and Table 2, respectively. Bacterial strains were routinely grown in Tryptic soy broth (TSB) medium (5 g/L Soy peptone, 17 g/L tryptone, and 20 g/L NaCl) at 28 °C with shaking at 160 rpm or on TSA plates. Chloramphenicol (Cm, 30 μg/mL) was added when required for strain selection.

### 2.2. Experimental Fish

A total of 200 orange-spotted groupers (*Epinephelus coioides*) obtained from a commercial fish farm in Zhanjiang, China, were used for the animal experiments in this work. The groupers, with a mean body weight of 80.5 ± 6.8 g and a mean body length of 15.5 ± 1.2 cm, were housed in 300-L opaque tanks (20 fish per tank) at 23–25 °C for two weeks. Before the experiment, sera from three fish were randomly extracted and tested against formalin-inactivated *V. alginolyticus* by slide agglutination. Fish that were negative in the sera agglutination and bacteriological recovery tests were tested as described by Pang et al. [19].

### 2.3. Cell Culture

The CIK cell line was purchased from the Cell Storage Center of Wuhan University (Wuhan, China) and cultured in L15 medium supplemented with 10% (*v*/*v*) FBS (Gibco, New York, NY, USA).

### 2.4. Cloning and Sequencing of the vopS Gene from V. alginolyticus HY9901

Primers were designed according to the *V. alginolyticus* gene sequence (GenBank Number: KX245316). PCR was carried out in a Thermocycler (Bio-Rad, Hercules, CA, USA) with the following ideal amplification conditions: 30 cycles of 95 °C for 30 s, 55 °C for 45 s, and 72 °C for 90 s after an initial denaturation at 95 °C for 5 min. The amplification product (5 μL) was examined on 1% agarose gels, stained with ethidium bromide. Additionally, the PCR products were converted into *E. coli* DH5α by ligating them to the pMD18-T vector (Table 1). DNA sequencing was performed by Sangon Biological Engineering Technology & Services Co., Ltd. (Shanghai, China). Similarity analyses of the nucleotide sequences and deduced amino acid sequences were performed according to the method of Zhou [18]. Additionally, ExPASy tools were used to analyze protein sequences (http://expasy.org/tools/), accessed on 21 May 2020.

### 2.5. Physiological and Pathological Characterization of ∆vopS

The Δ*vopS* phenotype was characterized by cell morphology, growth ability, extracellular protease (ECPase) activity, biofilm formation, swarming motility, and fifty percent lethal dose (LD_50_). In summary, the Δ*vopS* and the wild-type strain HY9901 were cultivated in TSB for 18 h, and scanning electron microscopy was used to observe the cell morphology. For growth ability analysis, bacteria were grown in TSB for 16 h, and then subcultured into fresh TSB (OD_600_ = 0.05) and cultured at 28 °C with shaking. ECPase activity was carried out using the previously described protocol [20]. Biofilm formation was measured using the previously published crystal violet stain method [21]. Swarming diameter was measured following a 24-h incubation period, and swarming motility was assessed using the methodology outlined by Zhou et al. [18].

The LD_50_ was determined according to the Tan’s method [22]. Briefly, 180 fish were haphazardly assigned to eighteen 150-L tanks (10 fish per group), each containing 120 L of sea water. The injection concentrations of HY9901, Δ*vopS*, and C-*vopS* were 10^4^, 10^5^, 10^6^, 10^7^, 10^8^, and 10^9^ cfu/mL. In the experimental group, 100 μL of bacterial solution was injected intramuscularly into each fish, whereas the control group received 100 μL of PBS. Mortalities of experimental fish were recorded over 14 days until the mortality rate stabilized. The morbidity induced by *V. alginolyticus* was determined, and the LD_50_ was calculated with three replicates per group.

### 2.6. Real-Time Quantitative PCR

The expression of T3SS-related genes was detected by real-time quantitative PCR. In brief, HY9901 and Δ*vopS* were cultured in DMEM medium for 12 h and were collected by centrifugation.

According to the experimental method of Li et al. [23], total RNA was extracted from all strains using TRIzol Reagent (Invitrogen, Carlsbad, CA, USA), and was subsequently reverse-transcribed into cDNA with Reverse Transcriptase M-MLV (Takara, Kyoto, Japan). RT-qPCR was carried out on a Roche LightCycler^®^ 96 System (Basel, Switzerland). The 16S rRNA gene was used as the internal control.

### 2.7. Hoechst 33258 Staining

Hoechst 33258 staining was used to identify apoptosis, following Burdette et al.’s instructions [24]. As controls, cells were either treated with 2 µM staurosporine or left untreated. A fluorescence microscope (Leica, Wetzlar, Germany) was used to detect the fluorescence signal at 450 nm.

### 2.8. Lactate Dehydrogenase (LDH) Release Assay

As previously mentioned, the *V. alginolyticus* strain was used to infect CIK cells. As controls, cells were either treated with 2 μM staurosporine or left untreated. As directed by the manufacturer, LDH release was quantified using a Cytotoxicity Detection kit (Jiancheng, Nanjing, China). 

### 2.9. Caspase-3 Activity Assay

Caspase-3 activity in CIK cells was detected using the commercially available caspase-3 assay kits (Jiancheng, Nanjing, China). Briefly, at 1, 2, and 3 h, the infected cells were collected and lysed using the cell lysis buffer for 15 min on ice. To measure caspase-3 activity, 50 µL of the supernatant was combined with 50 µL of 2× reaction buffer and 5 µL of 4 mM AC-DEVD-pNA substrate after centrifugation. The reaction mixtures were incubated at 37 °C for 4 h and were subsequently measured at OD_405_ in a microplate spectrophotometer.

### 2.10. Metabolomics Analysis

CIK cells were seeded at 1 × 10 cells/well in 6-well plates and were infected for 2 h with HY9901 (MOI = 50). To remove residual medium components, the cells were subsequently harvested and washed three times with ice-cold PBS. Then, 300 μL of prechilled 80% methanol was added to the samples, which were then vortexed on ice and swirled for 30 s. Following 6 min of sonification, they were centrifuged for one minute at 5000 rpm and 4 °C. After being freeze-dried, the supernatant was dissolved in 10% methanol. The solution was then added to the analysis of the LC–MS/MS system. Three biological duplicates of each sample were examined, and metabolite levels were normalized to total cell counts.

To perform peak alignment, peak selection, and quantification for every metabolite, Compound Discoverer 3.1 (CD3.1, Thermo Fisher, Waltham, MA, USA) was used to process the raw data files produced by UHPLC–MS/MS (Thermo Fisher, USA). The molecular formula was predicted using the normalized data based on fragment ions, additive ions, and molecular ion peaks. Peaks were then compared to the mzCloud (https://www.mzcloud.org/, accessed on 11 January 2023), mz Vault, and Mass List databases in order to acquire exact qualitative and relative quantitative results. The statistical software R (version R-3.4.3), Python (version 2.7.6), and CentOS (version 6.6) were used to conduct the statistical studies.

### 2.11. Intracellular GSH and GSSG Assay

Cellular GSH and GSSG were measured according to the method of Tao [25]. The cell samples were infected with the *V. alginolyticus* strain, as described above. After combining the cells with the protein removal reagent solution, the samples were quickly frozen and thawed twice in liquid nitrogen and a water bath at 37 °C. Following centrifugation at 12,000× *g* for 10 min, the supernatant was collected. GSH and GSSG in samples were detected by the GSH/GSSG assay kit (Beyotime, Shanghai, China).

### 2.12. Detection of Intracellular Iron Content

CIK cells were seeded in 12-well plates and cultured to approximately 70–80% confluence, followed by incubation with HY9901 (MOI = 50). Following the removal of the supernatant after 2 h, the cells were washed with PBS and digested into a single cell using a 0.25% trypsin solution. As directed by the manufacturer (Elabscience, Wuhan, China), the intracellular ferrous ion content was ascertained.

### 2.13. Statistical Analysis

To conduct statistical studies, GraphPad Prism (v8.0) was utilized. The Student’s *t*-test was used to establish the statistical significance of differences between HY9901 and Δ*vopS*. The data obtained from investigations of bacterial counts, swarming diameter, biofilm formation, cell adherence, and agglutination titers are displayed as X ± SD. Duncan’s test was used to assess group differences. When *p* < 0.05, the data was deemed statistically significant.

### 2.14. Biosecurity

The bacteria protocols were approved by the Biosecurity Committee of Guang Dong Ocean University (Zhanjiang, China).

## 3. Results

### 3.1. The V. alginolyticus vopS Shows Higher Conservation Within Vibrio spp.

**Figure 1:**
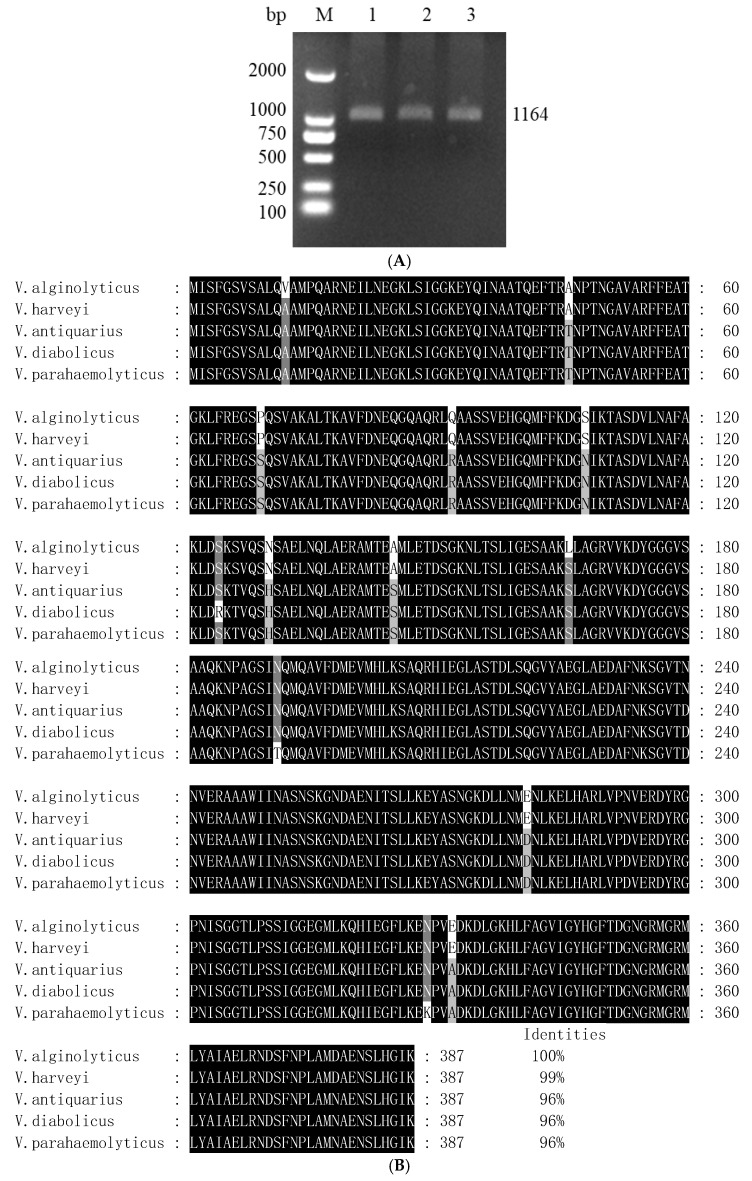
(**A**) Cloning of *vopS* gene. Lane 1: DL2000 marker. Lane 2–4: The 1164 bp fragment was amplified from genomic DNA of the wild-type strain HY9901 using primer pairs of vopS_1_/vopS_2_. (**B**) Homology comparison of the amino acid sequence of vibrio *vopS* with other bacteria *Vibrio alginolyticus* Accession No. WP_017821339.1; *Vibrio harveyi* group Accession No. WP_025768118.1; *Vibrio antiquarius* Accession No. WP_074191275.1; *Vibrio diabolicus* Accession No. WP_048626460.1; *Vibrio parahaemolyticus* Accession No. WP_053807485.1.

The nucleotide sequence analysis showed that the ORF of *vopS* was 1164 bp (GenBank: KX245316) (Figure 1A). The relative molecular mass is 41.47 kDa, and the isoelectric point is 5.51, as predicted by ExPASy. A BLAST analysis indicated that the deduced amino acid of *vopS* exhibits 96–99% identity with some Vibrio spp. In particular, it shared the highest homology to *vopS* of *V. harveyi* (Figure 1B).

**Figure 2:**
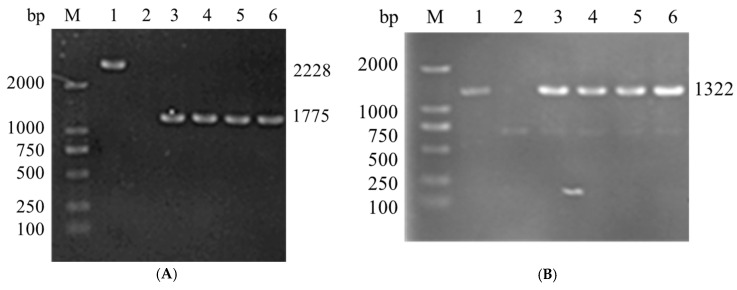
Construction and confirmation of the knockout mutant strain Δ*vopS* and C-*vopS*. (**A**) PCR identification of Δ*vopS* (Primers: *vopS*-TF/*vopS*-TR). Lane 1: DL2000 marker; Lane 2: HY9901; Lane 3: β2163 (pLP12-*vopS*); Lane 4–7: Δ*vopS*. (B) PCR identification of C-*vopS* (Primers: pBAD-mcf-TF/pNAD-mcf-TR). Lane 1: DL2000 marker; Lane 2: β2163 (*vopS*-pBAD33cm-rp4); Lane 3: Δ*vopS*; Lane 4–7: C-*vopS*.

Using overlap PCR and a double-selection approach, an unmarked *vopS* deletion mutant was created in order to comprehend the potential functions of *vopS* in *V. alginolyticus*. HY9901 was determined by PCR amplification by generating a fragment of 2228 bp, while Δ*vopS* was determined by PCR by generating a fragment of 1775 bp (Figure 2A). C-*vopS* was confirmed by PCR amplification by generating a fragment of 1322 bp (Figure 2B).

### 3.2. ΔvopS Had Weak Extracellular Enzyme Activity and Virulence Compared to HY9901

**Figure 3:**
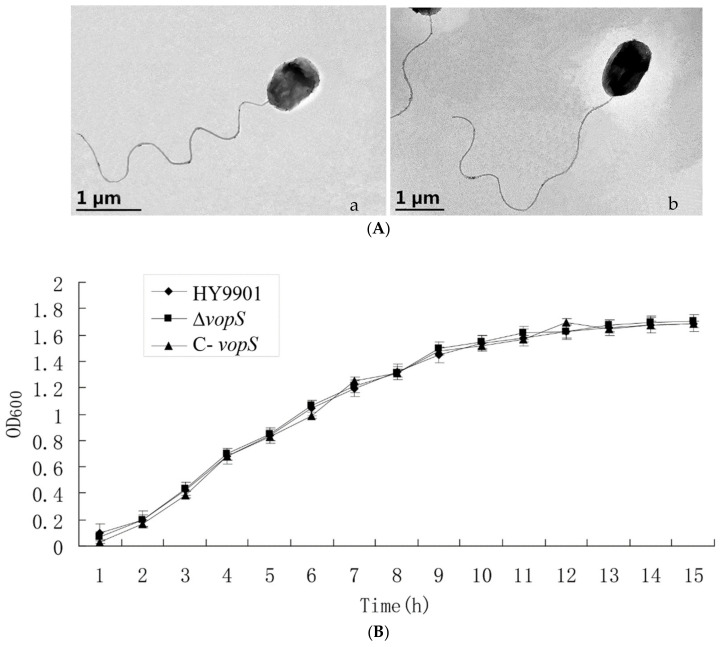

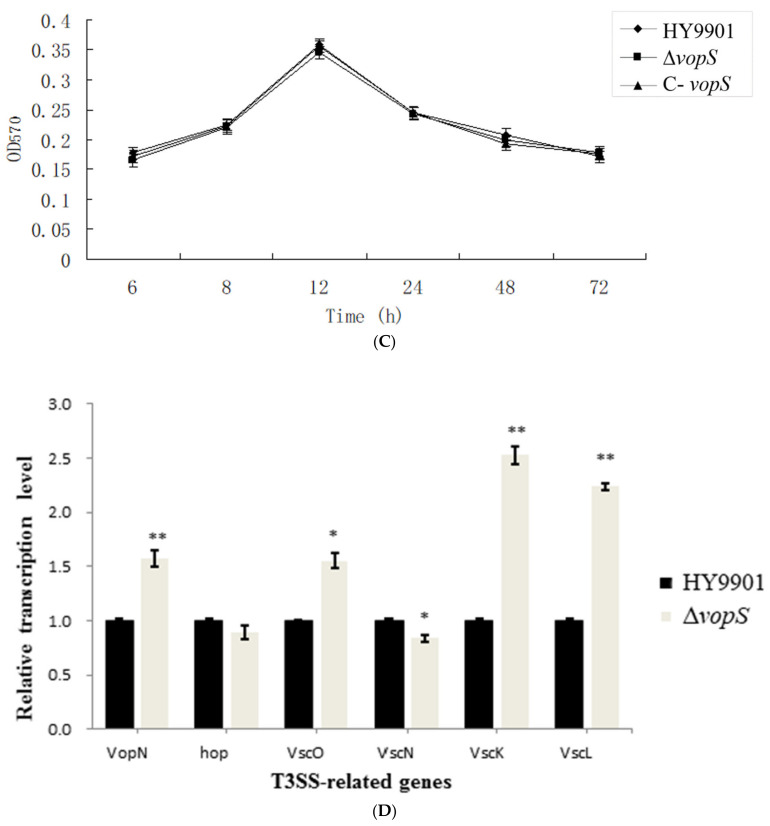
(**A**) Observation of the morphological feature of (**a**) HY9901 and (**b**) ∆*vopS* by SEM; (**B**) Growth curve of different strains; (**C**) Biofilm of different strains; (**D**) Expression of HY9901 and Δ*vopS* T3SS-related genes induced by DMEM. * indicates significant difference compared with the control group (*p* < 0.05). ** indicates extremely significant difference compared with the control group (*p* < 0.01).

In our preliminary experiments, we found that there was no discernible morphological difference between HY9901 and Δ*vopS* in transmission electron microscopy (TEM) (Figure 3A). Additionally, the deletion of *vopS* in HY9901 did not yield any differences in biofilm formation, growth, and adherence (Figure 3B,C). However, the extracellular protease activity of Δ*vopS* was significantly lower compared to HY9901(Table 3). To determine whether the *vopS* gene affected bacterial virulence, the orange-spotted grouper infectious disease model was used to determine the virulence in the three strains. The moribund fish and mortalities exhibited clinical symptoms of vibriosis, characterized by hemorrhaging, swelling, and ulcers on the skin surface. The LD_50_ value in HY9901 was significantly higher (6.29 × 10^5^ cfu/fish) than in the Δ*vopS* (3.43 × 10^7^ cfu/fish) (*p* < 0.01, Table 3), and the virulence of the complementary strain C-*vopS* was similar to that of HY9901. In conclusion, our results indicate that the *vopS* gene deletion reduced *V. alginolyticus* virulence.

### 3.3. VopS Affects the Transcription of T3SS Genes

VopS is an essential component of T3SS. To analyze the role of VopS in T3SS in depth, qRT-PCR was performed on the Δ*vopS*. Some T3SS-related genes were detected, including vsck, vscL, vscN, vopN, vscO, and *hop*. The *vscO* is a chaperone escort protein. The *vscN*, *hop*, and *vopN* are effector or regulatory, and *vscL* and *vscK* are apparatus proteins. The results showed that compared with HY9901, Δ*vopS* decreased the expression of *hop* and *vscN* and significantly increased the expression of *vscL*, *vscK*, *vopN*, and *vscO* (Figure 3D).

### 3.4. VopS Facilitates the Disruption of the Cytoskeleton and Induces Host-Cell Death

**Figure 4:**
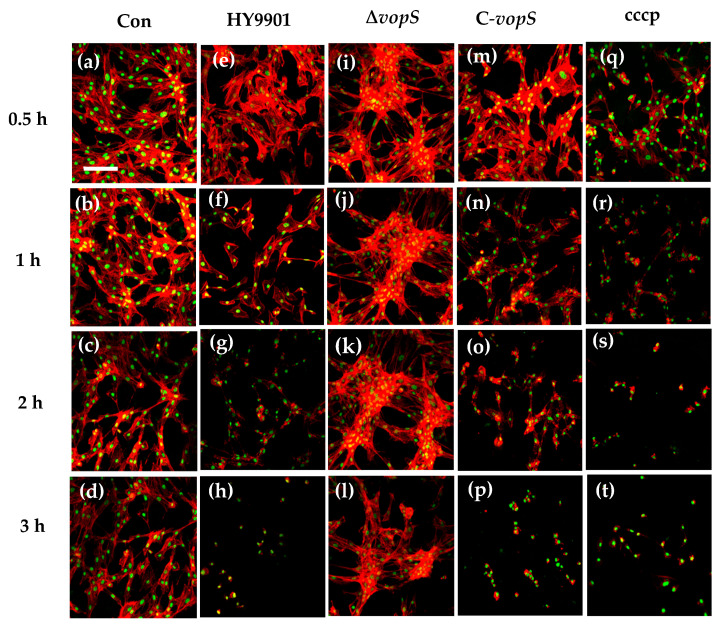
Laser scanning confocal microscopy infection observation (100 µm). *V. alginolyticus* infection of CIK cells induces nuclear condensation and fragmentation in a T3SS-dependent manner. CIK cells were infected with strain HY9901 (**e**–**h**), Δ*vopS* (**i**–**l**), or C-*vopS* (**m**–**p**) as described. Cells were treated with 2 μM STS as a positive control (**q**–**t**) or left uninfected(**a**–**d**) for the negative control. Nuclear condensation and fragmentation were visualized using Hoechst 33258 to stain nuclei at indicated time points after infection.

**Figure 5:**
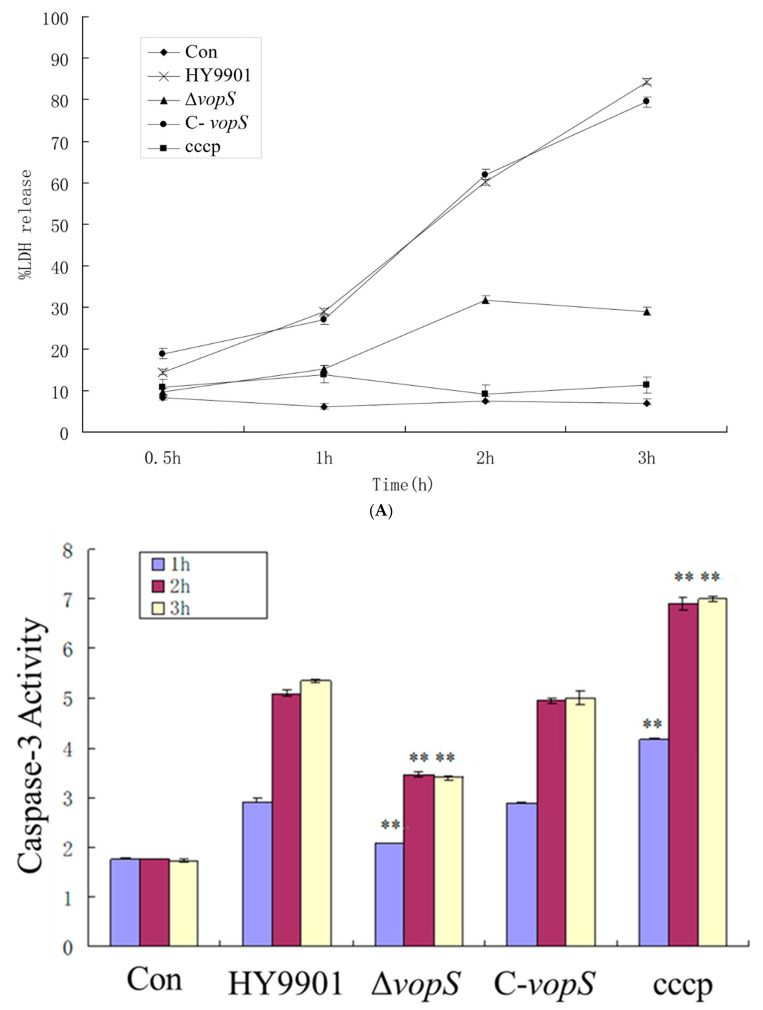
(**A**) LDH release caused by *V. alginolyticus* infection in CIK cells. (**B**) Measurement of caspase-3 activity. ** indicates extremely significant difference compared with the control group (*p* < 0.01).

Our previous results showed that *vopS* was the essential gene to regulate *V. alginolyticus* virulence. We then visualized the nuclear morphology using Hoechst staining in order to further describe the process of cell death caused by the T3SS of *V. alginolyticus*. Nuclear condensation was observed in some HY9901 and C-*vopS*-infected cells at 30 min post-infection (Figure 4e,m). As the infection time extended, increasing amounts of cells showed apoptotic characteristics, including nucleus fragmentation and formation of apoptotic body, which could be seen by fluorescence microscope (Figure 4f–h,n–p). This phenotype resembled that seen in carbony1 cyanide 3-chlorophenyl 1 hydrazone (CCCP), a strong inducer of apoptosis (Figure 4q–t). Conversely, the nuclear compartments in Δ*vopS*-infection cells remained intact during infection, which was roughly in line with the uninfected cells’ appearance (Figure 4i–l,a–d). An LDH release assay was conducted to further assess the cytotoxicity of Δ*vopS*. As early as 30 min after infection with the HY9901 and C-*vopS*, we noticed a notable release of LDH into the medium. LDH concentrations in the medium rose in tandem with the infection duration, reaching over 80% total LDH release 3 h after infection (Figure 5A). Nevertheless, there was no discernible difference in the levels of LDH between the medium of Δ*vopS*-infected and uninfected cells (Figure 5A).

It is well established that caspase-3 is highly relevant for apoptotic cell death. We next examined whether the activation of caspase-3 in infected cells results in apoptosis or pyroptosis as a result of *V. alginolyticus* infection. As shown in Figure 5B, caspase-3 in the HY9901 and C-*vopS*-infected cells was clearly synthesized after 1 h of infection and peaked at 3 h. There was no significant difference between the HY9901 and C-*vopS*. In contrast, the level of caspase-3 in Δ*vopS* mutant was significantly lower compared to HY9901. In conclusion, these results indicate that deletion of *vopS* reduces bacterial cytotoxicity.

### 3.5. V. alginolyticus Infection-Induced Distinct Metabolome Alteration in the Infected Host Cell

**Figure 6:**
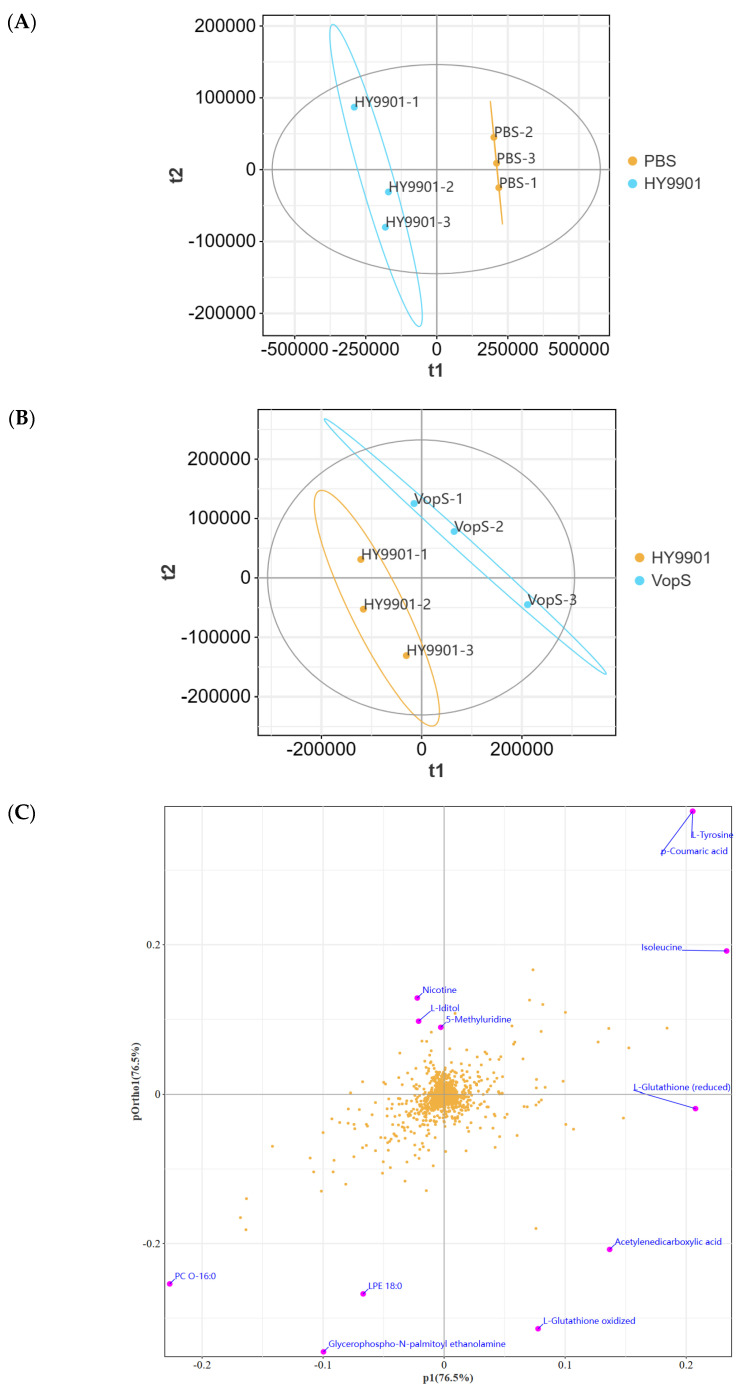

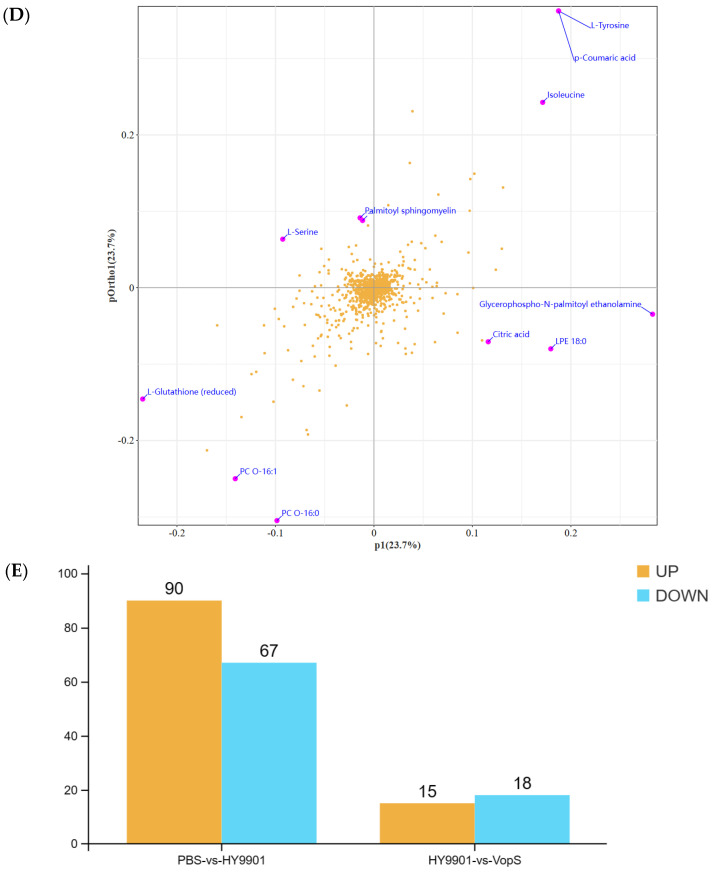

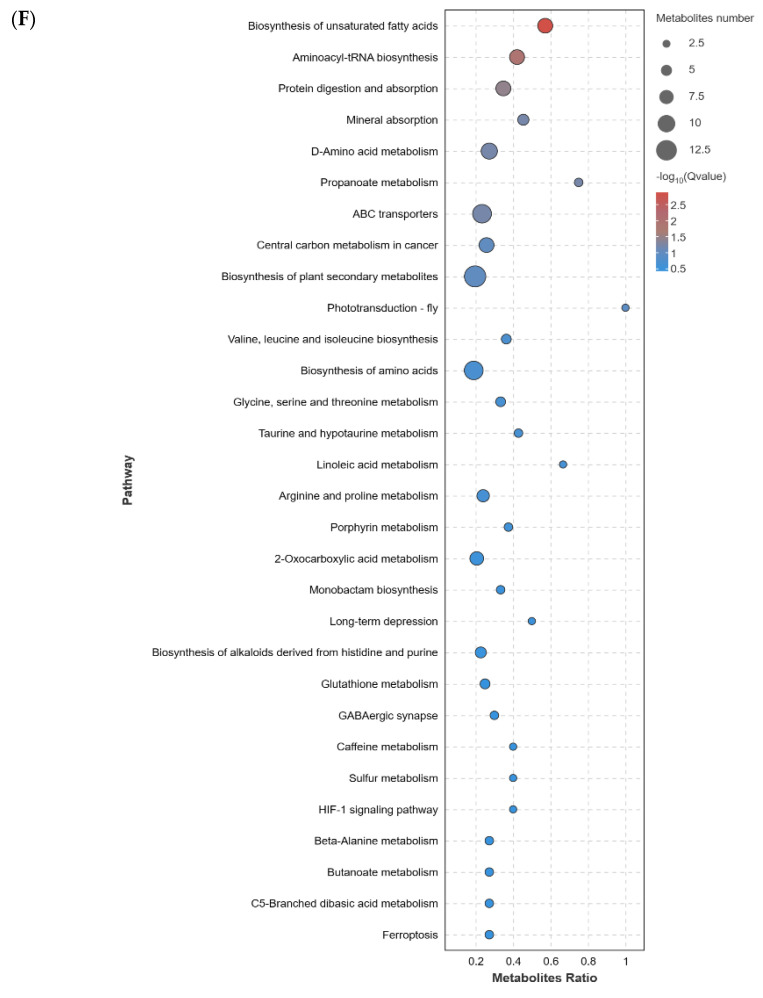

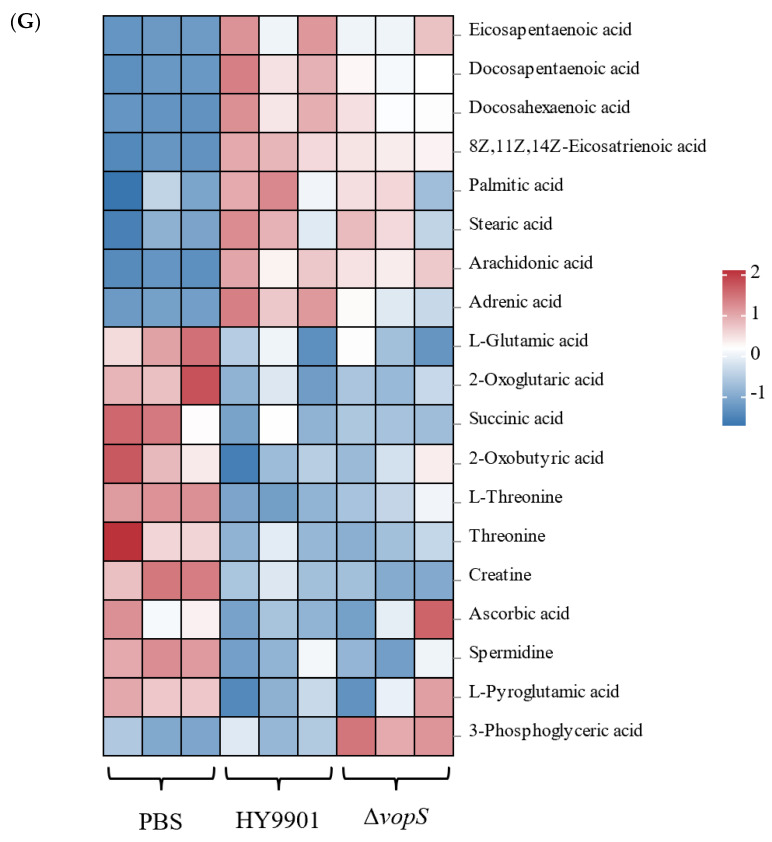

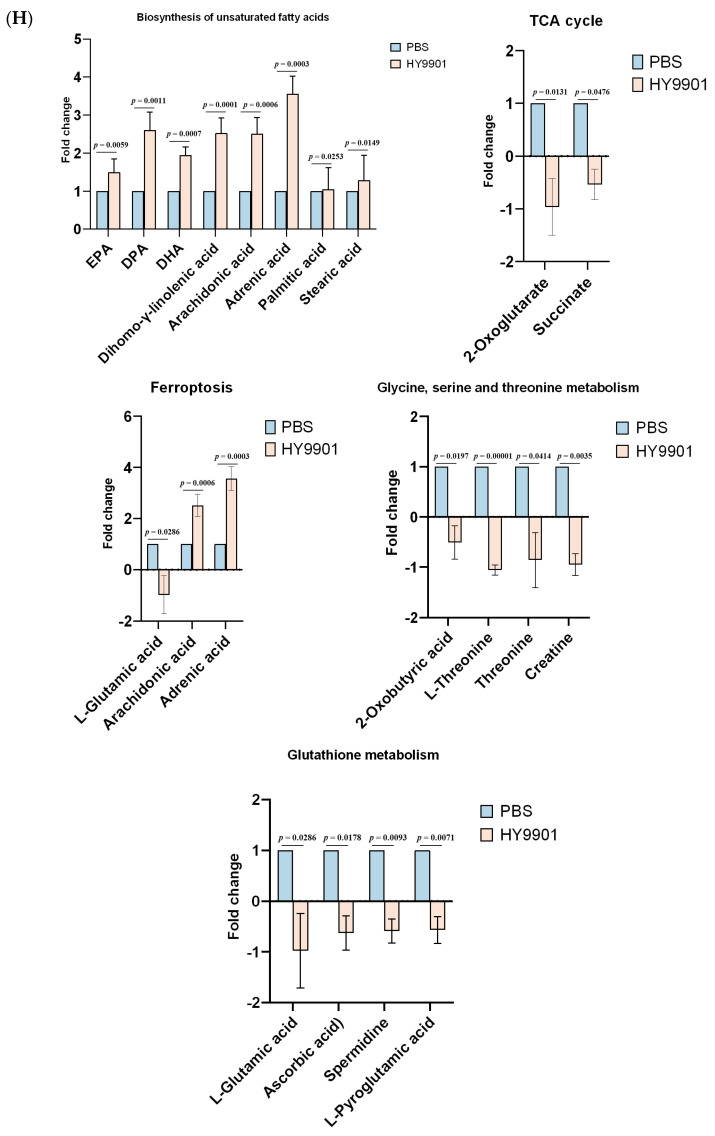
(**A**) Principal component analysis (PCA) of HY9901-infected group and non-infected group based on Bray–Curtis distance. (**B**) Principal component analysis (PCA) of HY9901-infected group and VopS-infected group based on Bray–Curtis distance. (**C**) S-plot generated from OPLS-DA for HY9901-infected group vs. non-infected group. (**D**) S-plot generated from OPLS-DA for HY9901-infected group vs. VopS-infected group. (**E**) Differential metabolites in PBS vs. HY9901 and HY9901 vs. VopS (*p* < 0.05, VIP ≥ 1). (**F**) KEGG showed 30 significantly (*p* < 0.05) enriched biological pathways associated with *V. alginolyticus* infection. (**G**) Significantly expressed metabolite categories related to *V. alginolyticus* infection and analyzed by heatmap. (**H**) Fold changes in biosynthesis of unsaturated fatty acids, TCA cycle, ferroptosis, glycine, serine, threonine, glycine, and glutathione metabolism for *V. alginolyticus* infection.

Bacterial virulence factors can be delivered to host cells to hijack cellular metabolism. To study the metabolomic responses of the CIK cell line to the infection of *V. alginolyticus*, untargeted metabolomics analysis was used to profile metabolite changes in the HY9901-infected cells, Δ*vopS*-infected cells, and non-infected cells. In the three group cells, 2250 metabolites were identified (Table S1). Partial least-squares discrimination analysis (PLS-DA) revealed that the metabolites in the three groups of cells clustered separately with no marked differences within groups (Figure 6A,B). Next, we used orthogonal partial least-squares discrimination analysis (OPLS-DA) to further screen for metabolites related to infection. Based on a correlation *p* ≥ 0.5 as a cutoff and an absolute covariance *p* ≥ 0.05, two S-plots (Figure 6C,D) were constructed and used to identify key metabolites or biomarkers in the three groups. When HY9901-infected cells and non-infected cells were compared, 157 differential metabolites were identified, with 90 upregulated and 67 downregulated (Figure 6E).

To define the impact of *V. alginolyticus* infection on host metabolism, further analysis of the metabolic pathways involved in the significantly altered metabolites using Kyoto Encyclopedia of Genes and Genomes, a pathway analysis tool, revealed that the top 25 enriched pathways were mainly enriched in eight categories: biosynthesis of unsaturated fatty acids, aminoacyl-tRNA biosynthesis, protein digestion and absorption, D-Amino acid metabolism, ABC transporters, central carbon metabolism in cancer, biosynthesis of plant secondary metabolites, and biosynthesis of amino acids (Figure 6F and Table S2).

Following KEGG enrichment results, we identified significant accumulations of biosynthesis of unsaturated fatty acids intermediates, including icosapentaenoic acid (EPA), docosapentaenoic acid (DPA), docosahexaenoic acid (DHA), dihomo-γ-linolenic acid, arachidonic acid, adrenic acid, palmitic acid, and stearic acid (Figure 6G,H). The levels of some tricarboxylic acid (TCA) cycle intermediates, including 2-oxoglutarate and succinate, were significantly decreased (Figure 6G,H). Notably, the levels of the tested ferroptosis intermediate L-glutamic acid were significantly decreased, while arachidonic acid and adrenic acid were significantly increased (Figure 6G,H). The levels of glycine, serine, and threonine metabolism and its downstream metabolites, glycine and glutathione (GSH), were significantly decreased in HY9901-infected cells (Figure 6G,H). Together, the metabolomics data indicate that *V. alginolyticus* infection enhances the biosynthesis of unsaturated fatty acids and ferroptosis, while reducing glycine, serine, and threonine metabolism and downstream metabolism of serine in infected CIK cells.

### 3.6. VopS Can Hijack Host Cell Fatty Acid Metabolism

**Figure 7:**
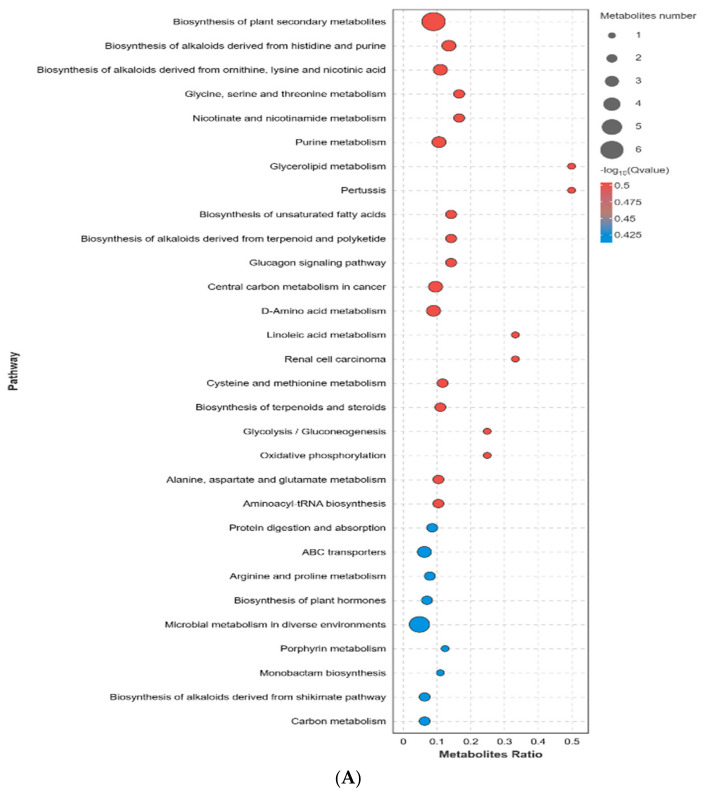

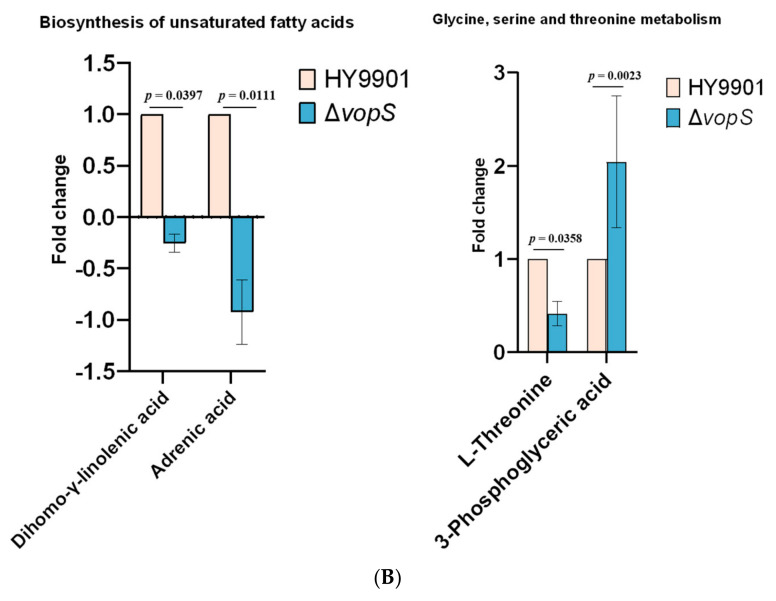
(**A**) KEGG showed 30 significantly (*p* < 0.05) enriched biological pathways associated with Δ*vopS* infection. (**B**) Fold changes in biosynthesis of unsaturated fatty acids and glycine, serine, and threonine metabolism for Δ*vopS* infection.

We then investigated the metabolic responses of the Δ*vopS*-infected cells. When Δ*vopS*-infected cells and HY9901-infected cells were compared, 33 differential metabolites were identified, including 15 upregulated and 18 downregulated (Figure 6E). KEGG enrichment analyses revealed that the top 25 enriched pathways were mainly enriched in two categories: biosynthesis of plant secondary metabolites and microbial metabolism in diverse environments (Figure 7A and Table S2). Since evidence from the above study indicated that *V. alginolyticus* is associated with the dysregulation of fatty acid metabolism in the host, we subsequently focused on fatty acid metabolites in Δ*vopS*-infected cells. The levels of some biosynthesis of unsaturated fatty acids intermediate, including dihomo-γ-linolenic acid and adrenic acid, were significantly decreased (Figure 6G and Figure 7B). However, in glycine, serine, and threonine metabolism, L-threonine and 3-phosphoglyceric acid were significantly increased (Figure 6G and Figure 7B). In conclusion, our results indicated that *V. alginolyticus* enhances the biosynthesis of unsaturated fatty acids and ferroptosis by secreting VopS into the host.

### 3.7. VopS Can Induce Ferroptosis of CIK Cells

**Figure 8:**
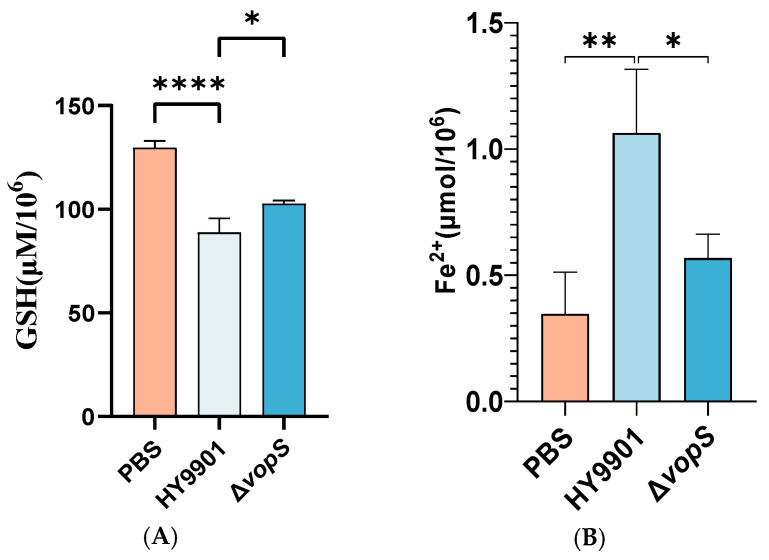
(**A**) Cellular glutathione concentrations of CIK cells infected with different *V. alginolyticus* mutants for 2 h. (**B**) Cellular Fe^2+^ accumulation of CIK cells infected with different *V. alginolyticus* mutants for 2 h. * indicates significant difference compared with the control group (*p* < 0.05). ** indicates extremely significant difference compared with the control group (*p* < 0.01). **** indicates significant difference compared with the control group (*p* < 0.0001).

Our previous studies have demonstrated that VopS promoted the accumulation of unsaturated fatty acids, especially the accumulation of adrenic acid. Adrenic acid was reported as a marker of ferroptotic cell death [26]. To confirm that VopS is involved in the induction of ferroptosis cell death, we detected the level of GSH and ferrous (Fe^2+^) in the infected cell. As shown in Figure 8A, *V. alginolyticus* HY9901 infection led to a remarkable depletion of GSH compared with the control group. Deletion of the *vopS* gene could ameliorate the *V. alginolyticus*-induced decrease in total GSH. We also found that the Fe^2+^ iron levels elevated after *V. alginolyticus* infection, and Fe^2+^ accumulation of Δ*vopS*-infected cells was strongly reduced compared to HY9901-infected cells (Figure 8B). Therefore, these results indicated that VopS could induce ferroptosis in CIK cells.

## 4. Discussion

Vibriosis encodes T3SS to deliver effector proteins directly into the host cell cytoplasm, enabling them to survive in an external environment and destroy various signaling pathways [27]. Thus, it is important to conduct further in-depth studies on T3SS in *V. alginolyticus* to illustrate its pathogenesis.

VopS is considered a main virulence factor of the *Vibrio parahaemolyticus* T3SS1 and induces macrophage apoptosis by NF-κB inhibition [28]. In this study, we cloned the *vopS* gene in *V. alginolyticus* HY9901. The *V. alginolyticus vopS* gene was highly conserved in vibriosis, suggesting that VopS was an important effector protein in *V. alginolyticus*.

Vibrio growth, biofilm formation, extracellular protease secretion, and motility play important roles in adaptability to the environment [29,30], which increases the probability of adhesion, colonization, invasion, infection, and, finally, pathogenic processes in the host. To comprehensively investigate the roles of VopS on the pathogenesis of *V. alginolyticus* HY9901, the ∆*vopS* strain was constructed. Compared to HY9901, there was no significant difference in morphology, growth, swarming, and biofilm formation in ∆the *vopS* strain, which resembled the studies of Zhou et al. and Chen et al. [31,32]. Interestingly, the extracellular protease activity of ∆*vopS* was decreased compared to HY9901, indicating that *vopS* may be a positive contributor to extracellular protease activity in *V. alginolyticus*. Furthermore, the fish infection assay indicated that *vopS* positively regulates the virulence of *V. alginolyticus* to *Epinephelus coioides*. These findings support the pathogenic role of VopS in *V. alginolyticus* infection.

The *V. alginolyticus* T3SS is a complex, highly regulated nanomachine, which is regulated by a single regulatory protein or multiple regulatory proteins. For example, the expression of *sycD*, *vopB*, and *vopD* is significantly decreased by T3SS apparatus protein gene *vscO* depletion compared with the *V. alginolyticus* wild-type strain [31]. As reported previously, the *vscX* mRNA level is significantly up-regulated in the late growth stage in Δ*vscO* [33]. Similar results have previously been reported in the study of Zhou et al. [18]. In this study, Δ*vopS* showed decreased expression of *hop*, which encodes T3SS effector protein, indicating that the decrease in virulence of Δ*vopS* is reasonable. Nevertheless, the expression of *vscL*, *vscK*, *vopN*, and *vscO* was increased in Δ*vopS*. These results suggested that *V. alginolyticus* has complex regulatory networks to control the expression of T3SS genes. The regulatory mechanism network is still unknown, and its role in pathogenesis warrants further exploration.

Apoptosis is a well-known mechanism of programmed cell death and is mechanistically mediated by caspases [34]. The Rho small G protein family members are considered to be involved in cytoskeleton regulation [35]. It was reported that *VopS* from *V. parahaemolyticus* induced apoptosis through guanosine triphosphatase inhibition [14]. We further observed the *V. alginolyticus*-infected cells using Hoechst 33258 and found that the cellular cytoskeletal structure of CIK cells clearly collapsed, confirming that this effect is driven by VopS-mediated apoptosis. CIK cells infected with HY9901 maintained nuclear membrane integrity, although nuclear condensation was observed in those cells. Notably, we observed the cytoskeletons gradually collapsed from 180 min post-infection but not from the initial 120 min post-infection in Δ*vopS* strain-infected cells, compared to the control results and HY9901 infection. It was previously known that caspase-3 is a critical effector enzyme involved in apoptosis [36]. In this study, we demonstrated that VopS contributes to the activation of caspase-3 and LDH. The *vopS* gene deficiency delays the onset of apoptosis, but still does not prevent it. There may be other factors involved in CIK apoptosis.

Previously, although several reports showed that vibriosis dramatically altered the tricarboxylic acid cycle (TCA), amino acid metabolisms, and host cell glycolysis [37,38], it was unclear which biochemical pathways of host cells were significantly altered by *V. alginolyticus*. We found that *V. alginolyticus* also repressed host cell energy metabolism, including TCA cycle and serine synthesis metabolisms. Importantly, we observed *V. alginolyticus* modulates CIK cell biosynthesis of unsaturated fatty acids metabolism to promote the accumulation of arachidonic acid and adrenic acid in host cells, which has been linked to ferroptosis. Ferroptosis is a type of iron-dependent cell death driven by an accumulation of lipid peroxides [39]. Ferroptosis and apoptosis share similar characteristics, and apoptosis is stimulated upon induction of ferroptosis [40]. In the last decade, scientists have discovered markers for determining ferroptosis. In addition to monitoring lipid peroxidation, altered GSH and Fe^2+^ content can serve as indicators of ferroptosis [41]. Herein, we detected the GSH and Fe^2+^ content in infected cells, and the results corroborated the presence of ferroptosis during *V. alginolyticus* infection. Among the metabolites determined by the LC–MS/MS system, we also observed that 1- to 2-fold decreased levels of biosynthesis of unsaturated fatty acids intermediate, such as adrenic acid and dihomo-γ-linolenic acid, in CIK cells infected by Δ*vopS* compared to *V. alginolyticus* HY9901 strains. Our data proved that deletion of the *vopS* gene could mitigate *V. alginolyticus*-induced GSH consumption and Fe^2+^ deposition of CIK cells, suggesting *vopS* could promote ferroptosis in host cells. Naturally, there are several limitations to our research. The current validation of ferroptosis has been performed only by GSH and Fe^2+^ contents determination. Further research is needed to explore the underlying mechanisms of VopS-induced ferroptosis.

## 5. Conclusions

Our work lighted on the significance of VopS in the *V. alginolyticus* infection and provided novel targets for therapeutic strategies against vibriosis. The *vopS* gene, which can promote virulence, is required for extracellular protease secretion. *V. alginolyticus* is able to reprogram the biosynthesis of unsaturated fatty acids metabolism of CIK cells to initiate sufficient accumulation of arachidonic acid and adrenic acid to induce ferroptosis. Also, it was demonstrated that VopS was involved not only *in V. alginolyticus*-mediated apoptosis but also in ferroptosis. Here, we reveal that VopS is a critical virulent effector in host-pathogen interaction.

## Figures and Tables

**Table 1:** Bacterial strains, plasmids, and cell lines used in this study.

Strains, Plasmids, Cell Line	Relevant Characteristics	Source or References
*V*. *alginolyticus* HY9901	Wild type, isolated from diseased *Lutjanus sanguineus* off the Southern China coast	This study
Δ*vopS*	HY9901 carrying an in-frame deletion of *vopS*	This study
*E. coli* DH5α	supE44 ΔlacU169 (φ80 lacZDM15) hsdR17 recA1 gyrA96 thi-1 relA1	Sangon, China
pBAD33-CM	araBAD promoter, Cmr	This study
pLP12	*E. coli*-suicide vector	This study
*E. coli* β2163	Competent cells	This study
β2163-pLP12-Δ*vopS*	β2163 containing plasmid of pLP12-Δ*vopS*, Cmr	This study
β2163-pBAD33-CM-Δ*vopS*	β2163containing plasmid of pBAD33-CM-Δ*vopS*, Cmr	This study
pMD18-T	Cloning vector, Amp^r^	Takara,J
pLP12-ΔvopS	pLP12 containing *vopS* gene in-frame deletion, Cmr	This study
CIK	Grass carp cells in the kidney	This study

**Table 2:** Sequences of primers used in this study.

Primer Name	Primer Sequence (5′-3′)
Cloning primers	
vopS_1_	ATGATCAGTTTTGGAAGTGTT
vopS_2_ mutant construction	TCACTTAATACCGTGAAGGCTA
*vopS*-MF1	GGAATCTAGACCTTGAGTCGACTTCTTTACTGACAGATTTTGCCA
*vopS*-MR1	TTACGAAGTTCTGCTATCGCGTATAGCGCGCTAACACTTCCAAAACT
*vopS*-MF2	AGTTTTGGAAGTGTTAGCGCGCTATACGCGATAGCAGAACTTCGTAA
*vopS*-MR2	ACAGCTAGCGACGATATGTCCTCTACGAGCAGAACATCGACAC
*vopS*-TF	CCATTTCTAAAATATTCACTGCCATAA
*vopS*-TR	GCACACCACCTGTTTCTCGAT
pLP-UF	GACACAGTTGTAACTGGTCCA
pLP-UR	CAGGAACACTTAACGGCTGAC
Complement construction	
pBAD30-ZF	CTAGAGTCGACCTGCAGGCA
pBAD30-ZR	AGCTCGAATTCGCTAGCCCA
vopS-RF	TGGGCTAGCGAATTCGAGCTAGGAGGAATTCACCATGATCAGTTTTGGA
vopS-RR	TGCCTGCAGGTCGACTCTAGTCACTTAATACCGTG
RP4-F2	CGAATTGGGTACCAGCGCTT
RP4-R2	TACCGTCGACGCCGGCCAGC
PBAD30-mcf-TF	CCATAAGATTAGCGGATCCTACCT
qPCR primers	CTTCTCTCATCCGCCAAAACAG
vscL-F	TACCACGGTGAGTGTAGTTC
vscL-R	CGTAACCGACTTCAGGGA
hop-F	CTTCGCTTTCGGTTTGCT
hop-R	AATACCATCCCACCCTGT
vscO-F	GAGCTGGAAACATTAAGACA
vscO-R	TTGCTGCAACTGAACGAA
vscK-F	GGCGTTATCTCCCGTTCC
vscK-R	CTCCGCCCACCATCAATA
vopN-F	TGAACTCGTTTCGGACTA
vopN-R	ACTTTCTGGACTCGCACT
vscN-F	TAGGCGAAGAAGGAATGG
vscN-R	GCGATAGAAGTGGCAACAA
YSCK-F	GGCGTTATCTCCCGTTCC
YSCK-R	CTCCGCCCACCATCAATA
16S-F	TTGCGAGAGTGAGCGAATCC
16S-R	ATGGTGTGACGGGCGGTGTG

**Table 3:** Characteristics of different strains.

Characteristics	HY9901	Δ*vopS*	C-*vopS*
Acitivity of ECP ^a^	0.47 ± 0.01	0.32 ± 0.01	0.47 ± 0.02
Swarming (mm) ^b^	44.7 ± 0.13	44.1 ± 0.17	44.6 ± 0.16
LD_50_ ^c^	6.29 × 10^5^	3.43 × 10^7^ **	6.35 × 10^5^

**: *p* < 0.01. Values are mean ± standard deviation for three trials. ^a^ Bacteria were incubated in TSB for 18 h at 28 °C. ^b^ Swarming diameters were measured after 24 h incubation on TSA containing 0.3% agar plates. ^c^ LD_50_ were evaluated in *E. coioides* with an average weight of 20.0 ± 2 g.

## Data Availability

The datasets supporting the conclusions of this article are available in MetaboLights with the unique identifier MTBLS10677 (http://www. ebi.ac.uk/metabolights/MTBLS10677, accessed on 23 May 2024) for the metabolomics dataset.

## Supplementary Materials

The following supporting information can be downloaded at https://www.mdpi.com/article/10.3390/ani14223250/s1, Table S1: All metabolites; Table S2: KEGG analysis.
